# Needs of Hemodialysis Patients and Factors Affecting Them

**DOI:** 10.5539/gjhs.v8n6p109

**Published:** 2015-10-20

**Authors:** Dhima Xhulia, Jaku Gerta, Zefaj Dajana, Ioannis Koutelekos, Chrysoula Vasilopoulou, Margitsa Skopelitou, Maria Polikandrioti

**Affiliations:** 1 Department of Nursing, TEI of Athens, Athens, Greece; 2 “Tzaneio” Hospital, Athens, Greece

**Keywords:** patients’, needs, hemodialysis, socio-demographic characteristics, clinical features

## Abstract

**Purpose:**

Of this study was to explore the needs of hemodialysis patients and the factors that affect them.

**Material & Methods:**

The sample of the study included 141 patients undergoing hemodialysis. Data collection was performed by the method of interview using a specially designed questionnaire which served the purposes of the study. The needs were grouped into six categories. Patients were asked to answer how important was for them each of the statements in the questionnaire. Furthermore, there were collected socio-demographic characteristics, information on health status and relations with the physicians and nurses, as well as data on the incidence of the disease in their social life.

**Results:**

The results of this study showed that patients evaluated as fairly important all six categories of their needs, with similar results in both sexes. Age was found to be statistically significantly associated with ’the need for support and guidance’, ’the need to be informed’ and ’the need to meet the emotional and physical needs’, (p=0.023, p=0.012, p=0.028 respectively). Education level was found to be statistically significantly associated with all patients’ needs with the exception of ’the need to trust the medical and nursing staff’, (p=<0.05). Place of residence was statistically significantly associated with ’the need for support and guidance’, (p=0.029). Furthermore, difficulties in relations with family members was found to be statistically significantly associated with ’the need for support, the need for communication and individualization of care’, (p=0.014, p=0.040, p=0.041). After multivariate analysis, however, it was shown that the only independent factor affecting ’the need for support and guidance’, ’the need for individualized care’ and ’the need to meet the emotional and physical needs’, was if the patients reported themselves as anxious or not (p=0,024, p=0,012 and p=0,004, respectively). In particular, patients who considered themselves anxious had 1.38, 1.5 and 1.6 points respectively higher score in the evaluation of the importance of needs compared to patients who did not consider themselves anxious.

**Conclusions:**

Factors affecting needs of hemodialysis patients are age, education level, place of residence difficulties in relations with family members as well as anxious personality as reported by the patients.

## 1. Introduction

Chronic kidney disease is an enormous public health problem, globally that is related to high morbidly and mortality (Lopez-Vargas, Tong, Sureshkumar, Johnson, & Craig, 2013).

It was estimated that during 2007 more than million individuals in the United States were affected by the disease while this number is about to double during the following ten years (Davison, 2007). Hemodialysis is the most common replacement therapy of renal function in patients with chronic kidney disease however it is a demanding therapy of high cost (Lopez-Vargas, Tong, Sureshkumar, Johnson, & Craig, 2013).

Hemodialysis patients experience a wide range of stressors that affect their daily life, such as loss of valuable personal time in dialysis sessions, uncertainty about the further outcome of the disease, loss of prior family and professional roles, long waiting for transplantation and finally dependence on both the machine and health professionals (Kimmel & Patel, 2006; Vasilopoulou et al., 2016).

Given that the management of hemodialysis demands patients’ awareness and participation in the therapeutic regimen, it is essential to enhance self care and promote autonomy with the ultimate goal to increase adherence and empower patients’ feel to survive.

During the last decades, there has been an increasing interest in understanding the needs of hemodailysis patients. Health professionals, specially nurses who are in proximity with patients spending time for their care, are able to plan a need-orientated approach which will permit patients to express themselves (Bayoumi & Alwakeel, 2012).

Furthermore, this approach will enable health professionals to provide holistic individualized health care.

The aim of the present study was to explore the needs of hemodialysis patients and the factors that affect them.

## 2. Material & Methods

### 2.1 Study Design

The study consisted of 141 patients (64 men and 77 women) undergoing hemodialysis in four dialysis centers. This sample was a convenience sample.

The study included all patients who met the entry criteria. Criteria for including a patient in the study were: a) good comprehension of Greek language and b) undergoing hemodialysis as a method of replacement of renal failure. Patients who met the entry criteria in the study were informed by the researchers verbally for the purposes of this study.

Then the researchers asked for the patients’ written consent for their participation. Only individuals who gave their consent were included in the study.

Data collection was performed by the method of interview using a questionnaire developed by the researchers in order to fully serve the purposes of the study.

The researchers reviewed the literature on the needs of hemodialysis patients and conducted informal interviews with patients and health professionals.

The data collected for each patient included: a) socio-demographic characteristics: gender, age, marital status, education level, place of residence and number of children, b) clinical characteristics: if the patient was suffering from any other illness, the degree of awareness of the health status, years since the onset of hemodialysis, the frequency and duration of hemodialysis, as well as information on how strictly they comply with treatment guidelines and the proposed diet and c) other information such as the relations with the physicians and nurses, the relations with the social and family environment, whether they concealed the problem from the social enviroment, if they reported themselves as anxious and if they had help at home.

Data collection lasted approximately 15 to 20 minutes.

The study was approved by the Medical Research Ethics Committee of each dialysis center and was conducted in accordance with the Declaration of Helsinki (1989) of the World Medical Association.

### 2.2 Evaluation of the Needs of Hemodialysis Patients

To evaluate the needs of hemodialysis patients, a questionnaire of 39 questions was used, regarding potential needs of patients, which has been used in previous research by Polikandtioti et al. (2011) in Greek population and both the reliability and the validity of the questionnaire had been tested.

Initially, this questionnaire was designed by Kristjandottir (1995) and had been translated in Greek by Kyritsi, Matziou, Perdicaris, & Evagelou (2005).

The researcher of the present study wished to explore the same needs in Greek hemodialysis patients. Furthermore, it was not found in literature review a similar tool to explore this wide range of needs in one questionnaire.

The statements were chosen in such way, so as to cover a wide range of hemodialysis patients’ needs. In fact, the statements were initially grouped based on their content in the following six categories of needs: a) the need to be informed, b) the need for guidance/support, c) the need to trust the medical and nursing staff, d) the need to communicate with doctors/nurses e) the need to cover emotional and physical needs of patients and f) the need for self-participation of patient’s care.

Patients were asked to answer how important they feel each of these statements was. A four degree scale, Likert type, was used to answer all questions. The 4 subdivisions representing the following answers: Not at all, Somewhat, Very and Very much.

The structural validity of the questionnaire (Polikandtioti et al., 2011) highlighted the following 6 factors:

- “the need for support and guidance”: consists of 9 questions.
- “the need to be informed from the medical and nursing staff”: consists of 8 questions.
- “the need for being in contact with other patient groups, and ensuring communication with relatives”: consists of 6 questions.
- “the need for individualized treatment and for the patient’s personal participation to his/her treatment “: consists of 6 questions.
- “the need to meet the emotional needs (eg, anxiety, fear, loneliness) and physical needs (such as relaxation, sleep, better conditions of treatment)”: consists of 7 questions.
- “the need to trust the medical and nursing staff”: consists of two questions.

Internal consistency test of these factors, showed that the internal consistency of the questions that make up each sub-scale is high (Cronbach’s a> 0.6), with the exception of the need to trust the doctors-nurses, where Cronbach’ a was found to be 0.549.

### 2.3 Statistical Analysis

Normality tests of continuous variables were performed, using the Kolmogorov-Smirnov test and histograms. All continuous variables in the study were not normally distributed, hence the analysis was performed using medians and not means. Nominal variables are presented using frequencies and percentages, whereas the continuous variables are presented with medians and inter-quartile range (25th-75th percentile).

The Kruskal-Wallis criterion was used to check the existence of association between the needs of patients’ and nominal variables with more than two categories. Bonferroni multiple comparisons were also performed. The Mann-Whitney criterion was used to check the existence of association between patients’ needs and nominal variables with two categories.

Multivariate linear regression was performed to investigate the independent factors associated with the level of patient needs. There was performed a multivariate linear regression model for each of the needs of patients (dependent variables). As possible factors (independent variables) were used all the features that found to be statistically significant associated at univariate level. The results are shown in β-coefficients and 95% confidence interval (CI) and p-value (significance).

Statistical significance was evaluated in 5% significance level. All statistical analyzes were performed with version 20 of the SPSS program (SPSSInc, Chicago, Il, USA).

## 3. Results

### 3.1 Patients’ Characteristics

Patients’ socio-demographic, clinical and other characteristics of the present sample are presented on Table 1.

**Table 1:** Descriptive information about the sample

Demographic characteristics	Ν	%
Sex (Females)	77	54.6
Age		
<40	14	9.9
41-50	28	19.9
51-60	34	24.1
61-70	39	27.7
71-80	26	18.4
Marital Status		
Married	62	44.0
Single	35	24.8
Divorced	11	7.8
Widow	33	23.4
Education		
Elementary	41	29.1
Secondary	54	38.3
BSc-MSc	46	32.6
Residency		
County capital	108	76.6
Rural Areas	33	23.4
No of children		
None	36	25.5
One	55	39.0
>=2	50	35.5
Clinical Characteristics		
Years from first hemodialysis		
Less than a year	13	9.2
2-5	59	41.8
6-10	51	36.2
>=11	18	12.8
Other disease (Yes)	90	63.8
Informed of the state of their health		
Very	8	5.7
Enough	86	61.0
Less	47	33.3
Followed the therapeutic doctor’s orders		
Very	14	9.9
Enough	80	56.7
Less/not at all	47	33.3
Followed the proposed diet		
Very	36	25.5
Enough	54	38.3
Less	36	25.5
Not at all	15	10.6
Frequency of hemodialysis		
3 times/week	141	100
Duration of hemodialysis (hours)		
3.00	25	17.7
3.50	31	22.0
4.00	85	60.3
Other characteristics		
Relations with nursing staff		
Very good	53	37.6
Good	72	51.1
Moderate	16	11.3
Relations with medical staff		
Very good	56	39.7
Good	57	40.4
Moderate/Bad	28	19.9
Relations with other patients		
Very good	74	52.5
Good	25	17.7
Moderate/Bad	42	29.8
Difficulties in relations with social environment		
Enough	19	13.5
Less	113	80.1
Not at all	9	6.4
Difficulties in relations with family environment		
Very/Enough	61	43.3
Less	47	33.3
Not at all	33	23.4
Conceal the problem from social environment (Yes)	61	43.3
Other person at home. who helps in everyday tasks (Yes)	104	73.8
Consider yourself anxious (Yes)	82	58.2

The majority of participating patients was 50-80 years old (70.2%) and married (44%). Regarding the place of residence, the vast majority of participants resided in a county capital (76.6%). Moreover, 41.2% of the sample underwent hemodialysis from 2-5 years. The majority of patients reported suffering from another disease (63.8%). More than half of the participants (61%) reported that they were enough informed of the state of their health.

66.6% of the participants followed quite/very closely the therapeutic doctor’s orders while 63.8% followed quite/very properly the proposed diet. All patients in the sample underwent hemodialysis 3 times/week and 60% of those hemodialysis lasted 4 hours.

The vast majority of the sample (88.7% & 80.1%) stated that they maintained good relation with the nursing staff and medical staff, respectively. Also, almost half of the patients (52.5%) stated that they maintained very good relations with other patients in the group.

Regarding the relations of patients with the social environment, only 13.5% reported that they faced many difficulties, while many more were those who reported having several or many difficulties (43.3%) in their relations with the family environment.

143.3% of the participants concealed the problem from the social environment, while the vast majority (73.8%) had someone at home who helped with daily activities. Finally, the majority (58.2%) reported themselves as anxious.

**Patients’ needs**

Table 2 represents the descriptives of the sub-scales of needs of hospitalized patients undergoing hemodialysis. From the median scores of sub-scales, it is concluded that patients rated the needs as important enough, given that the median scores of all sub-scales were close to the upper value of the range. Similar results were found in both sexes.

**Table 2:** Descriptive data of the sub-scales assessing the importance of the needs of patients undergoing hemodialysis

Patients’ needs (range)	Total score
*Need for support and guidance (9-36)*	24(20-25)
*Need to be informed from the medical and nursing staff (8-32)*	23(18.5-25)
*Need for being in contact with other patient groups, and ensuring communication with relatives (6-24)*	18(15.5-19)
*Need for individualized treatment and for the patient’s personal participation to his/her treatment (6-24)*	18(15-20)
*Need to meet the emotional needs (eg, anxiety, fear, loneliness) and physical needs (such as relaxation, sleep, better conditions of treatment) (7-28)*	20(16-21)
*Need to trust the medical and nursing staff (2-8)*	6(5-7)

Data are presented in medians (25th-75th percentile).

Association between socio-demographic characteristics and patient needs

From Table 3 it is showed that no socio-demographic characteristic was significantly associated with the patients’ need to trust the medical and nursing staff.

**Table 3:** Socio-Demographic characteristics and patients’ needs

Demographic characteristics	Support and guidance	To be informed from the medical and nursing staff	For being in contact with other patient groups, and ensuring communication with relatives	*For individualized treatment and for the patient’s personal participation to his/her treatment*	Meet the emotional and physical needs	Trust the medical and nursing staff
Sex						
Male	23(19-25)	22.5(17-25)	18(14.25-20)	18(14-19)	20(15-21)	6.5(5-7)
Female	24(20-20.5)	23(19.5-25.5)	18(16-19)	18(15-20)	19(16.5-21)	6(5-7)
Age						
<40	20.5(18-25)	21.5(17-24)	17(14-19)	17.5(13-20)	18(15.75-21)	5.5(5-7)
41-50	22.5(18.5-25)	22(18.24)	17(14-19)	16.5(14.25-19)	18(15.25-20)	6(5-7)
51-60	22(19-24.25)	21.5(17-24)	17(14.75-19)	16.5(14-19.5)	18.5(14-21)	6(5-7)
61-70	24(20-26)	24*(20-26)	19(16-19)	18(15-21)	20**(17-22)	7(6-7)
71-80	25*(22.75-25.25)	25*(21-26.25)	19(16-20)	19(16.75-21)	20**(17.5-21.25)	6.5(5-7)
Marital Status						
Married	23.5(20-25)	22(18.75-25)	17(15-19)	17.5(15-19)	19(16-21)	7(5-7)
Single	23(20-25)	23(17-26)	18(14-20)	18(13-20)	20(16-21)	6(5-7)
Divorced	24(16-26)	23(19-24)	17(16-19)	18(14-20)	20(16-22)	6(5-8)
Widow	24(20-26)	25(19.5-26.5)	18(16-19)	18(15-21)	20(16-21)	7(5-7)
Education						
Elementary	24(20.5-26)	24(18.5-27)	18(16-19)	18(14-21)	20(16-21)	7(5-7)
Secondary	24(20.75-26)	23.5(20-25)	19(16-20)	19(16-21)	21(17.75-22)	7(5-7)
BSc-MSc	21*,**(18-24)	21*,**(17-24)	16*,**(14-19)	15.5*,**(13.75-18)	17*,**(15-20)	6(5-7)
Residency						
County capital	23(19-25)	22(18-25)	17.5(15-19)	18(14-20)	19(16-21)	6(5-7)
Rural Areas	24***(22-26)	24(22-25)	19(16.5-20)	18(15-19)	20(18-22)	7(5-7)
No of children						
None	23(20-25)	23(17.25-24)	18(14-20)	18(13.25-19.75)	20(16-21)	6(5-7)
One	24(21-26)	24(20-26)	19(16-20)	18(15-20)	20(16-21)	7(5-7)
>=2	23(19-25.25)	22(17.75-25)	17(15-19)	17.5(15-19)	19.5(16-21)	6.5(5-7)

data are presented in medians (25th-75th percentile).

* p-value<0.05 for comparison with the first category of each factor, after Bonfferoni correction.

** p-value<0.05 for comparison with the second category of each factor, after Bonfferoni correction.

*** p-value<0.05 significant difference between the two categories.

Instead, it was found that age, education level and place of residence were significantly associated with patients’ need for support and guidance, (p=0.023, p= 0.004 & p=0.029). This need was more important to patients aged 61-80 years compared to all other younger patients, although the difference was statistically significant only in relation to patients <40 years. Regarding the level of education and place of residence this need was less important to BSc-MSc graduates compared with other patients, and individuals residing in a county capital in relation to persons residing in rural areas (Table 3).

The patients’ age and education level was found to be significantly associated with their need for information from the medical and nursing staff (p=0.012 & p=0.013, respectively). More specifically, this need was more important to patients aged 61-80 years compared to those aged <40 years and less important to BSc-MSc graduates compared with patients with lower educational level (Table 3).

Also, only level of education was significantly associated with the patients’ need for being in contact with other patient groups, and ensuring communication with relatives (p=0.001) and the need for individualized treatment and for the patient’s personal participation to his/her treatment (p=0.001). These needs were less important to people who were BSc-MSc graduates compared to other people (Table 3).

The need to meet the emotional and physical needs of patients undergoing hemodialysis was significantly associated with age (p=0.028) and the level of their education (p=<0.001). It seems that this need was more important to patients aged 61-80 years compared with those who were 41-50 years and less important to BSc-MSc graduates (Table 3).

### 3.2 Association between Clinical Characteristics and Patient Needs

No clinical characteristic was significantly associated with patients’ needs, hence the results are not presented in details in any Table.

### 3.3 Association between Other Characteristics and Patient Needs

Table 4 presents the results from testing the association between various patient characteristics and their needs.

**Table 4:** General sample characteristics and patients’ needs

Clinical characteristics	Support and guidance	To be informed from the medical and nursing staff	For being in contact with other patient groups, and ensuring communication with relatives	For individualized treatment and for the patient’s personal participation to his/her treatment	Meet the emotional and physical needs	Trust the medical and nursing staff
Relation with nursing staff						
Very good	23(18.5-25)	23(18-25.5)	18(15-19)	17(14-20)	18(15-21)	6(5-7)
Good	24(20.25-25)	23(19.25-25)	18(16-19)	18(15-19)	20(16.25-21)	6(5-7)
Moderate	24(17-26)	20.5(16-25.8)	18(14.5-21)	16.5(13.3-19.3)	19.5(15.3-21.8)	7(5-7)
Relation with medical staff						
Very good	22(18-25)	22.5(18-25)	18(14.25-19)	17(14-19)	19(13-21)	6(5-7)
Good	24(21-25.5)	23(19-25.5)	19(16-19.5)	19(15-20.5)	20(15-21)	6(5-7)
Moderate/Bad	24(20-26)	23(17.75-25)	17(15-19)	16.5(14.3-19.8)	20(13-21.3)	7(5.25-7)
Relation with other patients						
Very good	22(19-25)	22(18-25)	17.5(14.75-19)	17.5(15-19)	19(16-21)	6(5-7)
Good	23(21-25)	24(20-26.5)	18(17-20)	18(15-21)	20(16.5-21)	7(5-7)
Moderate/Bad	24(20-26)	23(20-26)	19(15.75-19)	18(14-20)	20(16-21)	7(5-7)
Difficulties in relations with family members						
Enough	24(20-26)	24(20.5-26)	18(16-19)	18(15-21)	20(15.5-21)	7(5-7)
Less	24(21-25)	23(20-25)	18(16-20)	18(14.25-19)	20(17-21)	6(5-7)
Not at all	21(18-25)	20*(17-23)	16*,**(14-19)	16**(13-19)	18(16-20)	6(5-7)
Conceal the problem from social environment						
Yes	24(20-26)	24(20.5-26)	19(16-20)	18(15-21)	20(16.5-21)	7(5-7)
No	22.5(20-25)	22(18-25)	17(14.3-19)	17(14.25-19)	19(16-21)	6(5-7)
Other person at home, who helps in everyday tasks						
Yes	23(20-25)	22(18.3-25)	17(15-19)	17(15-19)	19(16-21)	6(5-7)
No	24(21-26)	25(18.5-27)	19(16-20)	19(15-21)	21(16.5-22)	7(5-7)
Consider yourself anxious						
Yes	24(21-26)	24(20-26)	19(16-20)	19(15-21)	20.5(17-22)	7(5-7)
No	22***(18-25)	22***(17-24)	17***(14-19)	16***(14-19)	18***(15-20)	6(5-7)

Data are presented in medians (25th-75thpercentile).

* p-value<0.05 for comparison with the first category of each factor, after Bonfferoni correction.

** p-value<0.05 for comparison with the second category of each factor, after Bonfferoni correction.

*** p-value<0.05 significant difference between the two categories.

The characteristic that was significantly associated with the needs, apart from the need to trust the medical and nursing staff, was whether the patient considered himself anxious or not (p=0.001, p=0.005, p=0.025, p=0.001, & p=0.001, respectively for the other needs). What is concluded from the tables is that the patients, who did not characterize themselves as anxious, think of the needs to be less important.

Moreover, the difficulty in relations with family members was associated with the need to be informed from the medical-nursing staff, the need for being in contact with other patient groups, and ensuring communication with relatives and the need for individualized treatment and for the patient’s personal participation to his/her treatment (p=0.014, p=0.040, & p=0.041 respectively).

As shown in Table 4, patients who did not face any difficulties in their relations with the family environment they found less important these needs compared to patients facing many difficulties and few difficulties in their relations with the family.

### 3.4 Multivariable Analysis

A multivariable linear regression analysis was performed. In the model we introduced all the above statistically significant associations for each need separately, to explore whether the factors remain statistically significant.

From Table 5 it is shown that that after multivariable control, the only independent factor that remained statistically significant in terms of its association to the need for support and guidance was whether the patient considered himself anxious or not. Patients who considered themselves anxious had 1.38 points higher scores in how important they evaluated this need, than patients who did not characterize themselves anxious.

**Table 5:** Results of multivariable linear regression

Independent Factors	Support and guidance	To be informed from the medical and nursing staff	For being in contact with other patient groups, and ensuring communication with relatives	*For individualized treatment and for the patient’s personal participation to his/her treatment*	Meet the emotional and physical needs
**Age**	**β (95% CI)**	**β (95% CI)**	**β (95% CI)**	**β (95% CI)**	**β (95% CI)**
<40	ref. cat.	ref. cat.			ref. cat.
41-50	0.305 (-1.886-2.496)	-0.397 (-2.987-2.193)			-1.197 (-3.154 - 0.761)
51-60	-0.870 (-3.176-1.437)	-1.579 (-4.296-1.138)			-1.538 (-3.598 - 0.521)
61-70	0.241 (-2.512-2.994)	0.734 (-2.469-3.936)			-0.639 (-3.087 - 1.809)
71-80	1.457 (-1.475-4.390)	1.270 (-2.168 - 4.708)			-0.113 (-2.730 - 2.504)
**Education**					
Elementary	ref. cat.	ref. cat.	ref. cat.	ref. cat.	ref. cat.
Secondary	1.140 (-0.506 - 2.787)	0.582 (-1.328 - 2.492)	0.796 (-0.358 - 1.949)	0.697 (-0.509 - 1.903)	1.384 (-0.078 - 2.846)
BSc-MSc	-0.506 (-2.869-1.858)	-0.495 (-3.254-2.264)	-1.169 (-2.379 - 0.042)	-1.127 (-2.392 - 0.138)	-0.957 (-3.061 - 1.147)
**Residency**					
County capital	ref. cat.				
Rural Areas	1.009 (-0.387 - 2.405)				
**Difficulties in relations with family members**					
Enough		ref. cat.	ref. cat.	ref. cat.	
Less		-0.032 (-1.600-1.535)	0.752 (-0.353 - 1.857)	0.704 (-0.452 - 1.859)	
Not at all		-1.825 (-3.876- 0.226)	-0.476 (-1.917 - 0.966)	0.005 (-1.501 - 1.512)	
**Consider yourself anxious**					
No	ref. cat.	ref. cat.	ref. cat.	ref. cat.	ref. cat.
Yes	1.388 (0.185 - 2.591)	0.527 (-1.125 - 2.178)	0.586 (-0.559 - 1.730)	1.537 (0.340 - 2.733)	1.607 (0.537 - 2.677)

Regarding the need to be informed from the medical-nursing staff and the need for being in contact with other patient groups, and ensuring communication with relatives, there was no independent factor found statistically significant. For the need for individualized treatment and for the patient’s personal participation to his/her treatment, the factor whether the patient was anxious or not remains statistically significant. Patients who considered themselves anxious had 1.5 points higher score in how important they evaluated this need, than patients who did not consider themselves anxious.

Regarding the need to meet the emotional and physical needs, statistically significant factor was whether the patient was anxious or not, again. Specifically, patients who considered themselves anxious had 1.6 points higher score than patients who do not consider themselves anxious.

## 4. Discussion

The results of the present study showed that 43.3% had several or many difficulties within family environment.

Difficulty in relation with family environment was associated with: a) the need be informed from the medical-nursing staff, b) the need for being in contact with other patient groups, and ensuring communication with relatives, c) the need for individualized treatment and for the patient’s personal participation to his/her treatment.

Patients who did not face any difficulties with family environment considered these needs as less important. Therefore, one of the crucial issues in the implementation of effective holistic treatment to hemodialysis patients is to increase family involvement in the therapeutic regimen. Considering patients’ family as an integral part of the multidisciplinary group of health professionals, is obviously one of the most effective ways to enhance compliance to treatment (Tejada-Tayabas, Partida-Ponce, & Hernández-Ibarra, 2015; Ziegert, Fridlund, & Lidell, 2007).

Interestingly, families of hemodialysis patients have to accept a new reality due to various limitations imposed by the disease. According to Barnett et al., (Barnett, Li Yoong, Pinikahana, & Si-Yen, 2008) difficulties within family are mainly attributed to the extensive lifestyle changes including fluid and dietary restrictions. Additionally, frequent visits to dialysis centers as well as alteration between autonomy and dependence, deprive hemodialysis patients of prior roles or activities in daily life. Survival of patients and better outcome of the disease demand support by family members (Ahrari, Moshki, & Bahrami, 2014; Kara, Caglar, & Kilic, 2007; Zamanzadeh, Heydarzadeh, Oshvandi, & Lakdizaji, 2007; Aghakhani, Sharif, Molazem, & Habibzadeh, 2014) as well as a secure and stable environment for patients. It is equally important to train nursing staff to need-orientated approach with ultimate goal the long-term treatment success and patient’s adjustment to the illness (Miracle, 2005).

As it is shown by the results of the present study 43.3% concealed the problem from the social environment. A plausible explanation is that hemodialysis patients experience psychological distress when their weakness is obvious. Concealing health problem from the environment may reflect up to some extent social isolation and should be detected as it is related to high morbidity and mortality (House, 2001). On the other hand, social support promotes adherence to dietary and fluid restrictions while family support is highlighted as the highest of perceived support (Ahrari, Moshki, & Bahrami, 2014).

Statistical analysis of the data revealed that education level was associated with all patients’ needs with the exception of the need to trust the medical and nursing staff. Specifically, these needs were lower among participants who were graduates of tertiary education, possibly because they were more able to understand deeper the therapeutic guidelines. Another plausible explanation is that patients of tertiary education are able to to maintain a quality of everyday life, thus making less demanding the care provided by health professionals.

Age associated with: a) the need for support and guidance, b) the need for information from the medical and nursing staff, and c) the need to meet emotional and physical needs. These needs were more important to patients aged 61-80 years. Possibly, hemodialysis patients at this age apart from physical and cognitive impairment, they also experience insecurity, dependence on health professionals, feelings of loneliness and anxiety. Significantly more, treatment of patients at advanced age is more demanding since they have to face with other co-morbidities. It is not rare that their access to dialysis units is difficult as it may require wheelchair access or transportation by ambulance.

In support of this view, Cook & Jassal, (2008) claimed that hemodialysis patients over 65 years old experience functional impairment and disability. Lo, Chiu, & Jassal, (2008) stated that disability in hemodialysis patients is associated with hospitalization. More in detail, patients one week after discharge experienced deterioration in functionality however they still maintained the ability to use phone and settle their financial issues.

The only independent factor affecting the need for support and guidance, the need for individualized care and the need to meet the emotional and physical needs, was if the patients reported themselves as anxious or not.

Anxiety is the subjectively unpleasant feeling of dread over anticipated events, usually characterized as an overreaction to a state experienced as menacing. However, anxiety has a beneficial effect when it motivates individuals to act towards their good.

Hemodialysis patients reporting themselves as anxious usually perceive as a threat all the changes in daily life including restrictions imposed by the disease. Following this line of thought, it is possible that they experience insecurity about future, fear about the outcome of the disease or shortened lifespan. Another alternative suggestion is that patients characterizing themselves as anxious usually wish to exert control on the disease. Interestingly enough, they consider both the need for individualization of care and the need to meet the emotional and physical needs as important ones.

Christensen & Ehlers, (2002) stated that limitations accompanying hemodialysis affect the sense of control and therefore patients fail to develop adjustment to the disease. The ways hemodialysis patents perceive the disease seem to be of vital importance because according to Chilcot, Wellsted, & Farrington, (2011) it is a predictor of mortality.

Regarding the need for support and guidance, it is worth noting that the therapeutic patient-nurse relation passes through various stages. After diagnosis health professionals fight for patient’s survival and usually fail to provide psychological support. On the onset of hemodialysis, patients turn for support to the family, while in advanced disease they seek for help to medical and nurse staff so as to alleviate family daily stress.

However, it is intriguing to ascertain the critical role of providing support and guidance. Nurses who are sincere and open to hemodialysis patients during the sessions construct such a support that becomes a coping resource (Sturesson & Ziegert, 2014).

However, anxiety and needs of patients seem to be interactive. Anxiety increases patients’ needs but on the other hand unmet needs may increase anxiety. Moreover, diagnosis and treatment of anxiety in hemodialysis patients is often underestimated or delayed because the symptoms of anxiety are usually attributed to uremia (Feroze, Martin, Reina-Patton, Kalantar-Zadeh, & Kopple, 2010; Feroze, Martin, Kalantar-Zadeh, Kim, Reina-Patton, & Kopple, 2012).

Regardless of all the above explanations, such individuals merit further evaluation as according to Cukor et al. (2008) anxiety has a negative impact on quality of patients’ life.

## 5. Conclusions

Special strategies of early assessment and fullfilment of hemodialysis patients’ needs would have a positive effect on outcome of the isease since they help patients to clarify, plan and actualize their goals.

Enhancing awareness about anxiety and patients’ needs among nursing stuff would benefit hemodialysis patients.

**Limitations of the Study**

The present sample was a convenience one and not a representative of all hemodialysis patients.

This methodology limits the ability generalization of results.
